# Profile and characteristics of the adequacy of blood transfusions in Trauma Intensive Care. A cross sectional multicenter study

**DOI:** 10.3389/fpubh.2023.1133191

**Published:** 2023-03-20

**Authors:** Raúl Juárez-Vela, Manuel Quintana-Diaz, Antonio Rodríguez-Calvo, José Ángel Santos-Sánchez, María Gero-Escapa, Elena Gallego-Curto, Pedro José Satústegui-Dordá, Juan Luis Sánchez-González, Carlos Jericó, Regina Ruiz de Viñaspre-Hernández, Guadalupe Gil-Fernández, José Antonio García-Erce

**Affiliations:** ^1^Doctoral Program in Medicine and Surgery, Autonomous University of Madrid, Madrid, Spain; ^2^Department of Nursing, Research Group in Care (GRUPAC), University of La Rioja, Logroño, Spain; ^3^Center of Biomedical Research of La Rioja (CIBIR), Logroño, Spain; ^4^Intensive Care Unit, PBM Group, IdiPAZ, Hospital La Paz, Madrid, Spain; ^5^Complex Hospital University of Salamanca, Salamanca, Spain; ^6^Faculty of Medicine, University of Salamanca, Salamanca, Spain; ^7^Intensive Care Unit, Hospital of Burgos, Burgos, Spain; ^8^Intensive Care Unit, University Hospital of Cáceres, Cáceres, Spain; ^9^Research Group of the University of Zaragoza B43_20R Water and Environmental Health, Department of Physiatry and Nursing, Faculty of Health Sciences, University of Zaragoza, Zaragoza, Spain; ^10^Department of Nursing and Physiotherapy, Faculty of Nursing, University of Salamanca, Salamanca, Spain; ^11^Internal Medicine Department, Complex Hospitalari Moisés Broggi, Sant Joan Despí, Spain; ^12^Department of Nursing, University of Extemadura, Badajoz, Spain; ^13^Blood and Tissue Bank of Navarra, Servicio Navarro de Salud-Osasunbidea, Pamplona, Spain

**Keywords:** blood, transfusion, multiple trauma, blood transfusion, health policy

## Abstract

**Introduction:**

Major trauma is one of the major health care problems facing modern society, trauma systems require careful planning to achieve an ideal level of coverage for the population. The Patient Blood Management Program is an integrated and global strategy to provide patient care that aims to assess and address, when possible, the etiology of blood abnormalities rather than transfuse without treating the underlying cause. We aimed to describe the factors that are associated with the clinical decision to transfuse polytraumatized patients admitted to the Intensive Care Unit (ICU).

**Method:**

We performed a cross sectional multicenter study of patients admitted to ICUs for trauma in 14 Spanish hospitals from September 2020 to December 2021.

**Results:**

A total of 69 patients were treated in the emergency room due to polytrauma, 46% of them were considered serious in the initial triage. Thirty were caused by a fall from considerable height (43.47%), followed by 39 patients admitted due to trac accidents (56.52%). The location of the trauma was mainly cranioencephalic, followed by thoracic trauma. Of the 69 patients, 25 received a blood transfusion during their ICU stay (36.23%).

**Discussion:**

No significant differences were observed between transfused and non-transfused patients, except for the severity scales, where transfused patients have a higher score on all the scales assessed in the ICU except for the Revised Trauma Score. As we can see, the incidence of kidney failure was also different between the groups analyzed, reaching 44.00% in transfused patients and 13.64% in the group of patients without blood transfusion, *p* = 0.005. In this sense, 92.00% of the transfusions performed were inadequate according to the criteria of Hb in blood prior to the decision to transfuse (Hb < 9). Our data support the need to consider clinical practice guidelines regarding blood transfusion and its practices.

## 1. Introduction

Major trauma is one of the major health care problems facing modern society, trauma systems require careful planning to achieve an ideal level of coverage for the population (1). Blood management in critically ill patients is also of great importance, as the main cause of mortality in young adults is trauma, causing more than 5 million deaths a year worldwide (2, 3), with massive hemorrhage as the main cause of death (4). This makes evident the relevance of blood management in this type of patient.

Blood transfusion is one of the most commonly overused therapeutic interventions in the United States and the rest of the world. World Health Organization (WHO) strongly recommends the implementation of Patient Blood Management (PBM programs), whose main objective to promote adequate transfusion use, in concordance with the recent nationwide intermittent blood shortage, makes PBM more important than ever (5, 6). To reduce inappropriate transfusions, the Joint Commission accrediting agencies have proposed PBM performance indicators to promote restrictive transfusion practices. Many definitions of PBM have been given; however, the most widespread refers to a comprehensive and complete strategy to provide patient care that aims to evaluate and address the etiology of those anomalies, when possible, instead of promoting short-term therapy, such as a blood transfusion, without addressing the underlying cause (6).

PBM programs seek a multidisciplinary approach based on the care of patients who need a blood transfusion and to guarantee, at all times, the supply of blood and blood components when needed. Currently, this is a patient-centered approach that addresses such crucial issues as iron deficiency, anemia, coagulopathies, and blood loss, both in surgical and non-surgical patients. Anemia and iron deficiency are recognized as a serious problem global health problems that influence the quality of life (7). However, globally, there is still a gap in awareness of the implementation of PBM as a general framework to address the risks of iron deficiency, anemia, blood loss, and coagulopathy.

Restrictive transfusion practice is a key element of PBM, which advocates the principle of administering red blood cell (RBC) transfusions only when the benefits are considered to outweigh the risks, as well as to minimize the use of RBC units (8). Mortality in polytraumatized patients could be reduced through a multidisciplinary approach, carrying out an early diagnosis and treatment through goal-directed early hemostatic reconstitution. Although the risks of transfusing red blood cells are known and can be quantified, the short-term benefits are less certain and not easy to quantify. A 2016 Cochrane meta-analysis (9) of 32 trials in more than 12,000 patients compared restrictive transfusion with liberal RBC transfusion strategies. Patients randomized to restrictive transfusions were 43% less likely to receive RBC. The risk of dying within 30 days of RBC transfusion (primary outcome) was the same whether participants received red blood cells with lower or higher Hb levels. The objective of our study is to identify the factors that are associated with the clinical decision to transfuse polytraumatized patients admitted to the ICU, as well as the factors that are associated with the adequacy of the transfusion. It also aims to analyze the differences in the impact caused by adequate vs. inadequate transfusions on analytical values.

## 2. Materials and methods

### 2.1. Design

This was a cross sectional, prospective and multicenter study of patients admitted to intensive care units (ICUs) for trauma in 14 Spanish hospitals from September 2020 to December 2021. We include all the patients with a diagnosis of trauma admitted into the ICU. Due to movement restrictions imposed by the COVID-19 outbreak, admissions for trauma during the outbreak period were not excessive due to the restrictions traffic established by the government of Spain.

### 2.2. Variables and adequacy

The following information was collected through electronic medical records. Epidemiological variables: Age, sex, date of trauma and admission to the ICU, antiplatelet or anticoagulant drugs, and previous comorbidities were registered. We also used the registration of traumatic injuries according to the Abbreviated Injury Scale (AIS) (10) and the estimation of different severity indices, such as the ISS scale, TRISS (11), the revised trauma scale, and other more general scales used in intensive care units, such as the SOFA (Sequential Organ Failure Assessment Score), APACHEII (Acute Physiology and Chronic Health disease Classification System), and SAPS II (Simplified Acute Physiology Score) (12). All the applied scales allowed us to reliably measure the severity and have previously been validated for use in our setting. The adequacy of the transfusion was evaluated according to the pre-transfusion hemoglobin (Hb) levels and the characteristics of the patients, according to the 5th edition of the 2015 guideline on the transfusion of blood components and plasma derivatives of the Spanish Society of Blood Transfusion and cell therapy 2015, within the first 24 h and with the established cut-off of Hg < 9 g/dL. From a clinical point of view, the transfusion must be reevaluated taking into account clinical and analytical criteria. Likewise, it is currently recommended that the initial volume replacement be done with criteria of controlled hypotension to reduce bleeding and dilutional coagulopathy (13).

### 2.3. Data collection and sampling

A secondary analysis is done in which the data collection was carried out through the creation of an electronic data collection notebook. The information was treated confidentially and anonymously, since it was dissociated data, following the Regulation of Data Protection (EU) 2016/679 of the European Parliament and the Spanish Law. The study design was approved by the Ethics Committee of La Paz (PI 4155).

The convenience sampling was carried out in ICUs located in Madrid (La Paz), Cantabria (Marqués de Valdecilla), Asturias (Central Hospital of Asturias), the Basque Country (Donostia Hospital), Aragón (Clinico Lozano Blesa Hospital), La Rioja (San Pedro) Balearic Islands (Hospital Son Espasses) Canary Islands (University Hospital of the Canary Is-lands) Murcia (Hospital Virgen de la Arrixaca) Andalusia (Hospital Virgen del Rocio) Castille and Leon (Hospital de Burgos) Castille-La Mancha (Hospital de Guadalajara) and Catalonia (Germans Trias i Pujol). All patients who met the following inclusion criteria were selected for the study: those over 16 years old who had been admitted to the ICU with a diagnosis of trauma.

### 2.4. Statistical analysis

Quantitative variables are described using the mean and standard deviation or median and interquartile range according to the type of distribution. The normality of the distribution was analyzed using the Shapiro–Wilks test. Categorical variables are expressed *via* their frequency distributions and percentages. To analyze the differences between the categorical variables of the two groups, a chi-square test was used, or Fisher's exact test if the hypotheses of applicability of the former were not met. If the variables were quantitative, we used Student's *t-*test or the Mann–Whitney U-test according to the normality criteria. Statistical analysis was performed with the STATA/SE v.21.0 program (College Station, Texas, United States), with any value of *p* < 0.05 being considered statistically significant.

## 3. Results

### 3.1. Description of the sample

A total of 69 patients were classified in the emergency room due to polytrauma, 46% of them were considered serious in the initial triage. Thirty caused by fall from considerable height (43.47%), followed by 39 patients admitted due to traffic accidents (56.52%). The location of the trauma was mainly cranioencephalic, followed by thoracic trauma Of the 69 patients, 25 of them received a blood transfusion during their ICU stay (36.23%).

### 3.2. Factors associated with transfusion

Table 1 shows the demographic characteristics, clinical history, and severity scales at the time of admission to the ICU, as well as the prognostic variables concerning stay and death. No significant differences were observed between transfused and non-transfused patients, except for the severity scales, where transfused patients have a higher score on all the scales assessed in the ICU except for the Revised Trauma Score, as we can see (*p* = 0.539).

**Table 1:** Demographic characteristics.

		**No transfusion**		**Transfusion**		***P*-Value**
*N*		44	%	25	%	
Age (mean ± ST)		49.77 ± 20.37		44.84 ± 17.35		0.283
Gender	Men	32	72.73	20	80.00	0.500
	Women	12	27.27	5	20.00	
Diabetes	No	38	86.36	21	84.00	0.789
	Yes	6	13.64	4	16.00	
Hypertension	No	31	70.45	17	68.00	0.831
	Yes	13	29.55	8	32.00	
Dyslipidemia	No	35	79.55	20	80.00	0.964
	Yes	9	20.45	5	20.00	
COPD	No	41	93.18	24	96.00	0.630
	Yes	3	6.82	1	4.00	
Ischemic heart disease	No	43	97.73	25	100.00	0.448
	Yes	1	2.27	0	0.00	
Renal insufficiency	No	43	97.73	23	92.00	0.262
	Yes	1	2.27	2	8.00	
Anticoagulation	No	40	90.91	25	100.00	0.120
	Yes	4	9.09	0	0.00	
Antiaggregating	No	41	92.18	25	100.00	0.183
	Yes	3	6.82	0	0.00	
^*^SOFA-score (mean ± SD)		3.56 ± 3.00		6.24 ± 4.48		0.013^*^
^**^APACHE II (mean ± SD)		12.73 ± 7.31		19.80 ± 13.05		0.022^*^
^***^SAPS II (mean ± SD)		30.82 ± 15.67		44.72 ± 18.80		0.004^*^
Trauma score revised (mean ± SD)		7.17 ± 1.53		6.52 ± 2.21		0.539
ISS^∧^ (mean ± SD)		15.76 ± 13.18		36.04 ± 25.25		**<** 0.001^*^
ICU stay days (mean ± SD)		7.02 ± 8.13		13.24 ± 16.06		0.145
Hospital stay days (mean ± SD)		15.79 ± 10.09		24.39 ± 19.92		0.177
Mortality	No	38	88.37	21	87.50	0.916
	Yes	5	11.63	3	12.50	

* SOFA Scale, Sequential Organ Failure Assessment Score.

** Apache, Acute Physiology and Chronic Health Disease Classification System.

*** Simplified Acute Physiology Score II; ^∧^ Injury Severity Score.

Regarding admission to the ICU, the values of blood pressure, respiratory rate, body temperature, and CVP at admission were similar in both groups, as can be seen in the following table. In the care received in the ICU, we can highlight that 18 patients (72.00%) in the transfused group had orotracheal intubation compared to 19 patients (44.19%) in the non-transfused group, the difference being statistically significant (*p* = 0.026). The incidence of kidney failure was also different between the groups analyzed, reaching 44.00% in transfused patients and 13.64% in the group of patients without blood transfusion, p = 0.005 (see Table 2).

**Table 2:** Clinical characteristics.

**Vital signs**		**No transfusion**. ***N*** = **44**		**Transfusion**. ***N*** = **25**		***P*-Value**
		**Value (mean)**	**Range**	**Value (mean)**	**Range**	
Systolic BP^*^ on ICU^**^ admission		133	115–146	120	109–140	0.198
Diastolic BP on ICU admission		79	68–85	75	62–82	0.253
Temperature on admission to the ICU		36	35.5–36.5	36	35.0–36.3	0.082
CVP^***^ on admission to the ICU		6	4–10	7.5	7.0–9.0	0.484
Oxygen saturation on admission to the ICU		99	97–100	100	98–100	0.046^*^
Breathing frequency		18	16–20	18	14–22	0.848
**Clinical features**		**N**	**%**	**N**	**%**	***P*** **-Value**
Orotracheal intubation	No	24	55.81	7	28.00	0.026
	Yes	19	44.19	18	72.00	
Laryngeal mask	No	37	97.37	20	86.96	0.111
	Yes	1	2.63	3	13.04	
High flow oxygen therapy	No	37	90.24	22	88.00	0.774
	Yes	4	9.76	3	12.00	
Non–invasive mechanical ventilation	No	36	94.74	23	95.83	0.845
	Yes	2	5.26	1	4.17	
Invasive mechanical ventilation	No	24	57.14	11	44.00	0.298
	Yes	18	42.86	14	56.00	
Tracheotomy	No	39	97.50	19	86.36	0.088
	Yes	1	2.50	3	13.64	
Kidney failure	No	38	86.36	14	56.00	0.005^*^
	Yes	6	13.64	11	44.00	
**Days mechanical ventilation**		**No transfusion** ***N*** = **44**		**Transfusion**. ***N*** = **25**		***P*** **-Value**
		**Days (mean)**	**Range**	**Days (mean)**	**Range**	
		5	1–10	1	1–9	0.303

* BP, Blood Pressure.

** ICU, Intensive Care Unit.

*** CVP, Central Venous Pressure.

The values of the biochemical markers in both groups were not very different, as we can see in the following (Table 3). It should be noted that hemoglobin was somewhat lower in transfused patients where the median reached 11.10 (IR: 10.00–13.60) compared to values that rose to 13.00 (IR: 11.25–14.30), *p* = 0.028, as well as hematocrit where the median in transfused patients was 33.00 (30.10–40.60) compared to 38.60 (33.00–42.90) in non-transfused patients, *p* = 0.025. Significant differences were also found in fibrinogen, with smaller values in the group of patients who received blood transfusion (*p* = 0.009), pH where the transfused patients had slightly lower values, although statistically significant (*p* = 0.015), and the value of Glucose we highlight as significative as well (*p* = 0.005).

**Table 3:** Laboratory profiles.

**N**	**No transfusion**		**Transfusion**		***P*-value**
	**44**		**25**		
	**Value (mean)**	**Range**	**Value (mean)**	**Range**	
Leukocytes (Unit/mm^3^)	10,900	7,650–14,770	11,400	8,740–17,300	0.407
Hemoglobin (g/dl)	13.00	11.25–14.30	11.10	10.00–13.60	0.028^*^
Hematocrit (%)	38.60	33.00–42.90	33.00	30.10–40.60	0.025^*^
Platelets (Unit/mcL)	167,500	220–215,000	146,000	98,800–181,000	0.987
Fibrinogen (mg/dl)	330	250–400	240	171–298	0.009^*^
pH	7.40	7.30–7.40	7.30	7.30–7.30	0.015^*^
PO2 (mmHg)	95	68–140	157	86–233	0.143
PCO2 (mmHg)	40	37–45	43	38–49	0.134
Glucose (mg/dl)	135.00	105.50–168.00	174.50	152.50–192.00	0.005^*^
Creatinine (mg/dl)	0.80	0.60–1.00	1.00	0.80–1.30	0.015
Urea (mg/dl)	28.00	20.00–45.00	37.50	27.00–44.50	0.155
^*^K (mEq/L)	4.00	3.60–4.20	4.50	4.10–4.90	0.002^*^
^**^GPT (IU/L)^∧^	34.00	26.00–97.00	43.50	22.00–121.00	0.931
^***^GGT (IU/L)^∧^	36.00	16.50–72.00	58.00	23.00–73.00	0.530
Chlorine	105.00	104.00–109.50	107.00	102.50–110.50	0.643

* K, Potassium.

** GPT, glutamic pyruvic transaminase.

*** GGT, Gamma glutamyl transpeptidase

∧ International units per liter.

Factors associated with transfusion practice (adequate vs. inadequate transfusion).

The 25 patients who received a blood transfusion were classified into two groups, according to whether or not it was appropriate according guidelines. In this sense, 92.00% of the transfusions performed were inadequate according to the criteria of Hb in the blood before the decision to transfuse (Hb < 9) (14). There were no significant differences in transfused blood components, fresh frozen plasma, platelets, or red blood cells between the two groups, neither were there concerning the drugs administered, even though none of the drugs studied were applied in patients with transfusion adequacy. It should be noted that 30.43% of the patients without adaptation to transfusion received fibrinogen and 43.48% tranexamic acid.

Regarding comorbidities, significant differences were found in COPD and kidney failure, basically present in patients with adequate transfusion, as we can see in Table 4.

**Table 4:** Adequate vs. inadequate transfusion and comorbidities.

	**No Adequacy** ***N*** = **23**		**Adequacy** ***N*** = **2**		**Fisher**
	**N**	**%**	**N**	**%**	
Diabetes	3	13.04%	1	50.00%	0.300
Hypertension	7	30.43%	1	50.00%	0.547
Dyslipidemia	4	17.39%	1	50.00%	0.367
COPD	0	0.00%	1	50.00%	0.080
Ischemic heart disease	0	0.00%	0	0.00%	Na
Renal insufficiency	1	4.35%	1	50.00%	
Anticoagulation	0	0.00%	0	0.00%	Na
Antiaggregation	0	0.00%	0	0.00%	Na

Same patient may have more than one comorbidity.

Na, not applicable.

The differences observed in the analytical indices before the transfusion are notable. The patients who underwent an adequate transfusion showed values of Hemoglobin, Hematocrit, and platelet that are significantly lower than patients with inadequate transfusion, and values of leukocytes, urea, and GPT that are significantly higher.

Regarding Effect of the transfusion (adequate vs. inadequate) on the patient's analytical values no differences were found in the hemoglobin values in any of the groups; however, we can see in the following table how patients with inappropriate transfusion see their values lowered, from 11.60 (IR: 10.20–13.80) to 11.20 (IR: 9.90–13.70). Conversely in patients with transfusion adequacy, the hemoglobin values slightly increased from 7.25 (IR: 6.90–7.60) to 8.50 (IR: 7.90–9.10), although the difference is not significant, probably due to the sample size of said cluster. It should be noted that leukocytes decreased significantly in the inappropriate group, and values such as fibrinogen or potassium increased after the transfusion in the inappropriate group (see Table 5).

**Table 5:** Effect of the transfusion (adequate vs. inadequate) on the patient's analytical values.

	**Not Adequacy (*****n*** = **23)**				***P*-value**	**Adequacy (*****n*** = **2)**				***P*-value**
	**Pre-transfusion**		**Post-transfusion**			**Pre-transfusion**		**Post-transfusion**		
	**Value (mean)**	**Range** ^*^	**Value (mean)**	**Range** ^*^		**Value (mean)**	**Range** ^*^	**Value (mean)**	**Range** ^*^	
Leucocytes (Unit/mm3)	11,400	8,300–18,000	7,645	4,940–8,760	0.008^*^	13,610	9,920–17,300	8,745	8,600–8,890	0.500
Hemoglobin (g/dl)	11.60	10.20–13.80	11.20	9.90–13.70	0.712	7.25	6.90–7.60	8.50	7.90–9.10	0.500
Hematocrit (%)	33.40	31.00–40.80	32.80	30.8–40.00	0.921	21.50	20.00–23.00	24.70	22.40–27.00	0.500
Platelets (Unit/mcL)	14,6000	98,800–22,000	15,6000	104,000–178,000	0.510	123,000	77,000–169,000	160,500	77,000–244,000	1.000
Fibrinogen (mg/dl)	236.50	171.00–297.00	272.00	233.00–419.00	**<** 0.001^*^	445	445–445	605	605–605	1.000
Glucose (mg/dl)	174.50	152.00–192.00	188.00	(150.00–241.00)	0.092	172.00	160.00–184.00	(190.50)	184.00–197.00	1.000
Creatinine (mg/dl)	1.00	0.80–1.30	1.00	0.80–1.30	0.061	2.10	1.50–2.70	2.10	1.50–2.70	1.000
Urea (mg/dl)	36.50	25.00–42.00	37.00	30.00–42.00	0.152	49.00	41.00–57.00	50.50	41.00–60.00	1.000
K (mEq/L)	4.50	4.10–4.90	4.80	4.50–5.40	0.001^*^	(4.35)	4.10–4.60	4.75	4.70–4.80	0.500
GPT (IU/L)^**^	43.50	23.50–97.50	44.50	25.00–115.50	0.937	1,048.00	14.00–2,082.00	1,050.00	19.00–2,082.00	1.000
GGT (IU/L)	61.00	19.50–94.50	60.00	14.00–116.00	0.875	23.00	23.00–23.00	33.50	23.00–44.00	1.000

* Range of values within the group.

** (International units per liter).

## 4. Discussion

In this work, we have identified the factors that are associated with the practice of transfusing in Spanish ICU services. These factors have worse scores on the scales that measure the patient's clinical status, especially on the SOFA and Apache II scales; and SAPS II, differences in analytical parameters (lower hematocrit, Hb, fibrinogen, and arterial pH, and higher blood glucose and potassium levels), and the greater presence of kidney failure in transfused patients compared to non-transfused patients.

Regarding the factors that are associated with an adequate decision to transfuse, two factors have been identified: renal failure and COPD. By measuring the impact that transfusion produces on patients and analyzing the differences between those who have an adequate indication vs. those who have not found it, which statistically had no significant differences in transfused blood components, fresh frozen plasma, platelets, or red blood cells between the two groups. We found that the only criteria in non-adequate of 92.00% of blood transfusions are related to hemoglobin cut-off criteria, previous to the decision on transfusion (Hb < 9) (13). Transfusion is a common procedure in hospitals, especially in the Emergency Department and ICU, given the prevalence of anemia and bleeding in admitted patients.

The indication of transfusions must be carried out according to the references in the current guidelines, and it is necessary to carry out an individual assessment of each patient since blood components are a very scarce resource and their use must be limited to specific indications. Based on this, numerous associations have developed clinical practice guidelines to mitigate the problem of over- transfusion, reducing the number of inappropriate transfusions and optimizing the use of donated blood components (13, 14).

Regarding the adequacy of one transfusion, accumulating evidence suggests a lack of efficacy with red blood cell (RBC), plasma, and platelet transfusion in most critically ill patients. Evidence has also increasingly exposed previously known transfusion risks. A result is a growing number of recommendations for more restrictive, PBM-based red blood cell, plasma, and platelet transfusion strategies. An important exception to a more conservative transfusion practice occurs in patients with major trauma and life-threatening bleeding, such as the patients in our study. In these trauma patients, delaying therapies with red blood cells, plasma, and platelet components in this population can promote the lethal triad of coagulopathy, acidosis, and hypothermia with consequent increased bleeding, increased transfusion needs, and increased mortality (15).

As we can see in our study, one piece of information draws our attention, and it is that the decrease in hemoglobin and hematocrit, after a transfusion, leads us to hypothesize that they are patients with active bleeding. However, the criteria for blood transfusion, especially in trauma patients, are not according to the adoption of guidelines; for this reason, restrictive policies, with the exception of trauma, must be taken into account to avoid over- transfusion. With these policies, more recent studies (16) show that there is a notable improvement in the adequacy of RBC transfusion and a reduction in the overall utilization of red blood cells without affecting patient safety. All of this could be due to the implementation of patient blood management (PBM) programs.

Such programs minimize RBC transfusion and improve patient outcomes worldwide, as shown in their study by Shin et al. (17), where they conclude that implementing a PBM program, through a multidisciplinary clinical community, increased the appropriateness of RBC transfusion in the medical and surgical departments. According to the blood component transfusion guidelines (13, 14, 18), there are two types of recommendations we could recommend as suitable: Recommendation 1: A transfusion threshold of 70 g/L or below, with a target Hb range of 70–90 g/L, should be the default for all critically ill patients unless specific co-morbidities or acute illness-related factors modify clinical decision-making. Grade 1 B. Recommendation 2: Transfusion triggers should not exceed 90 g/L in most critically ill patients. Grade 1 B. Our results indicate that only 8% of the patients who received transfusion were adequate according to blood hemoglobin criteria before the decision to transfuse (19, 20).

Likewise, there were no differences in transfused blood components, fresh frozen plasma, platelets, or red blood cells between both groups (adequate vs. inadequate), nor concerning the drugs administered. Díaz et al. (21), in a cohort of 908 transfused patients where only 21.4% were adequately transfused, with statistically significant differences according to indication, hospital level, and prescribing physician.

Compared with these results, our analysis has shown approximately a percentage of inappropriate transfusions of 91%. The study carried out in Northern Ireland by Barr et al. (22) demonstrated that 23% of the transfusions were considered inappropriate with a sample size of *n* = 1,474, taking into account the difference in the sample and the limitations of our study, our results could hypothesize that in hospitals there is still an over-transfusion in patients, in concordance with other studies (23–29). These over-transfusions can seriously compromise their safety, exposing patients to the risk of serious transfusion reactions and unwanted situations cardiac overload (26) situations that could be improved with the development of patient blood management programs.

## 5. Limitation and strengths

Our study has many strengths, such as the fact that it is a multicentric study, and it assesses many variables that have allowed us to be aligned with international studies with similar characteristics. It also has a series of limitations. In the first place, due to the descriptive nature of the study, we cannot establish cause-effect relationships with which we have hypothesized situations by interpreting analytical data. Furthermore, the sample size is small, taking into account that during the COVID-19 restrictions, there were fewer patients suffering from trauma Finally, we establish the adequate vs. inadequate classification based only on clinical values, keeping in view that other factors can be taken into account.

## 6. Conclusion and implication for the clinical practice

Our data supports the need to follow the clinical practice guidelines for transfusions since blood is a limited resource. Because of these results, we recommend that prescribers be more aware of the need to prescribe transfusions of packed red blood cells appropriately to reduce over-transfusion and adverse reactions in the patient as is described in evidence.

## Data availability statement

The raw data supporting the conclusions of this article will be made available by the first author upon reasonable request.

## Ethics statement

The study design was approved by the Ethics Committee of La Paz (PI 4155). The patients/participants provided their written informed consent to participate in this study.

## Author contributions

RJ-V, MQ-D, and JG-E: conceptualization. RV-H: methodology. AR-C: software. JS-S: validation. RJ-V: formal analysis. MG-E: investigation. EG-C: resources. PS-D: data curation. RJ-V and JS-G: writing—original draft preparation. RV-H and RJ-V: writing—review and editing and funding acquisition. CJ: visualization. MQ-D and JG-E: supervision. JLS-G and GG-F: project administration. All authors have read and agreed to the published version of the manuscript.
